# The First In-Silico Simulation of Evolut-in-Evolut TAVR: Reproduction of a Real Clinical Scenario

**DOI:** 10.1007/s12928-025-01183-w

**Published:** 2025-08-11

**Authors:** Benedetta Grossi, Ottavia Cozzi, Giulia Luraghi, Damiano Regazzoli, Gianluigi Condorelli, Francesco Migliavacca, Giulio Stefanini

**Affiliations:** a Department of Biomedical Sciences, Humanitas University, Via Rita Levi Montalcini 4, 20072 Pieve Emanuele, Milan, Italy; b Department of Chemistry, Materials and Chemical Engineering, Politecnico di Milano, Piazza L. da Vinci 32, 20133 Milan, Italy; c IRCCS Humanitas Research Hospital, Via Alessandro Manzoni 56, 20089 Rozzano, Milan, Italy

**Keywords:** TAVR, TAV-in-TAV, Patient-specific simulations, Image-based computational simulations, Finite element method

## Abbreviations

TAVR: Transcatheter Aortic Valve Replacement

## Introduction

As transcatheter aortic valve replacement (TAVR) expands to younger patients, redo TAVR poses peculiar challenges, necessitating meticulous pre-operative planning. Predictive computational simulations may optimize device selection and positioning, potentially reducing complications.

An 86-year-old patient underwent a TAV-in-TAV procedure for a significant residual leak following a TAVR with a 31 mm Evolut CoreValve (Medtronic, USA). A second 31 mm CoreValve prosthesis was successfully implanted.

**Figure 1:**
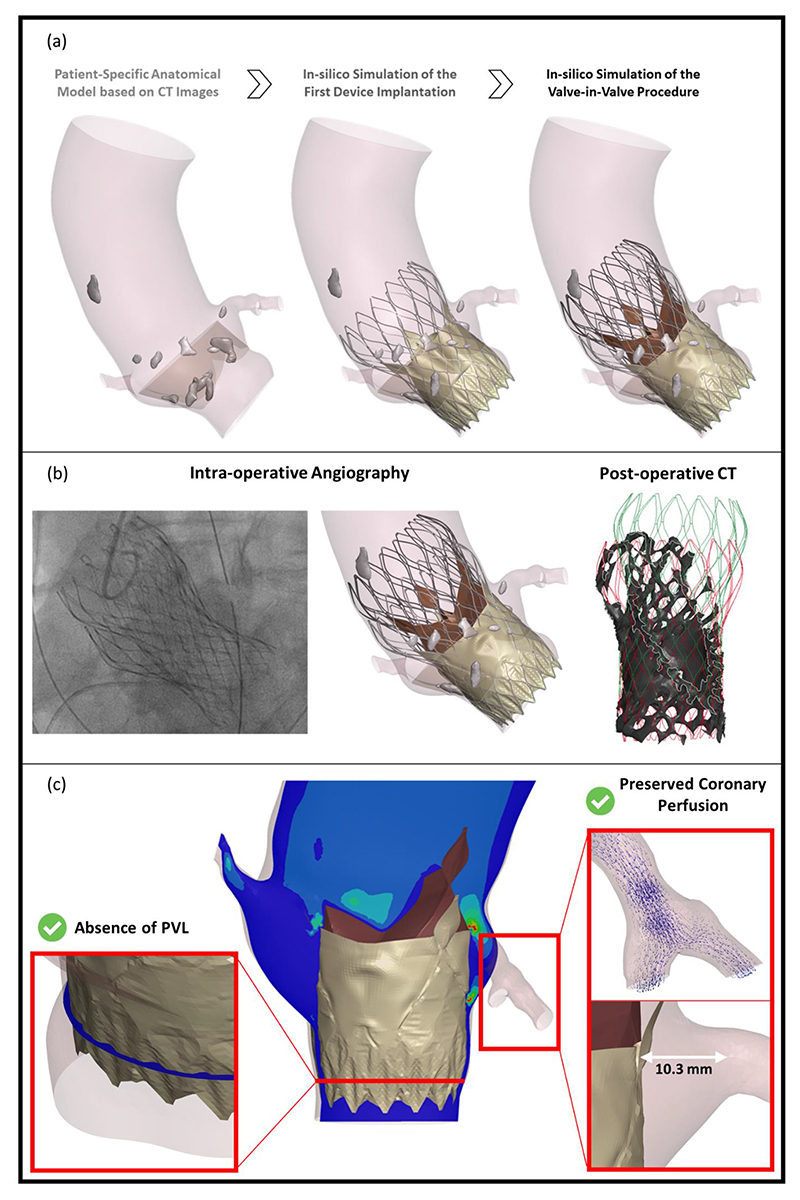
**a** Representation of the three main steps of the simulation workflow; **b** comparison of the simulated prosthesis configuration with intraoperative angiography and post-operative CT; **c** CFD simulation results showing absence of PVL and preserved coronary perfusion

Our clinically validated computational framework developed to virtually replicate TAVR in patient-specific anatomies [1] was adapted for this TAV-in-TAV procedure (Fig. 1a). This is the first in-silico simulation reproducing a redo TAVR with two Evolut prostheses. The patient-specific anatomy was reconstructed from CT scans. The virtual prostheses were positioned in the aortic root, and the simulation was then validated against post-operative imaging (Fig. 1b), confirming its accuracy. In addition, a post-implantation computational fluid dynamics (CFD) simulation was performed to evaluate post-operative paravalvular leakage (PVL) and coronary obstruction. Although retrospective, this analysis provided valuable procedural insights, notably highlighting reasons for suboptimal implantation outcomes, such as prosthesis underexpansion, as well as confirming absence of residual PVL and coronary obstruction after the second implant (Fig. 1c). The potential of prospective simulations lies in supporting device choice and placement strategies to improve outcomes. These patient-specific simulations allow testing of various devices, sizes, and orientations to identify the optimal approach.

Despite the need for multi-patient validation studies to confirm its robustness, this case exemplifies how image-based computational simulations can enhance procedural planning for complex TAVR cases, presenting a novel tool to refine outcomes in high-risk situations. Compared to conventional CT-based planning, simulations provide a more comprehensive assessment by accounting for mechanical interactions and dynamic blood flow, enabling precise prediction of outcomes such as PVL and coronary obstruction. Specifically, the simulations allow detailed evaluation of the post-implantation position of the displaced bioprosthetic leaflets relative to the coronary ostia, presented through three-dimensional visualization. Furthermore, CFD analyses quantify coronary flow rates, offering functional insights.

Importantly, the proposed workflow is designed for easy integration into clinical practice. Using standard contrastenhanced pre-operative CT scans, the process delivers a comprehensive simulation report within 2 working days. This report may include multiple implantation scenarios with comparative metrics that directly support the selection of the optimal device and positioning strategy. Notably, our workflow is flexible and readily adaptable to various clinical scenarios and valve types, making it well suited for broader application.
